# MDMA for the treatment of misophonia, a proposal

**DOI:** 10.3389/fpsyt.2022.983285

**Published:** 2022-11-10

**Authors:** Jadon Webb, Shannon Keane

**Affiliations:** ^1^Bloom Mental Health LLC, Littleton, CO, United States; ^2^Yale Child Study Center, New Haven, CT, United States

**Keywords:** MDMA, PTSD, misophonia, trauma response, psychedelic

## Abstract

Misophonia is a disorder characterized by negative physical and emotional reactions to certain trigger sounds, such as chewing food. Up to 50% of population samples endorse some symptoms of misophonia, with about 20% having symptoms that impair normal life functioning. Most misophonia patients exhibit intense negative emotions and autonomic arousal (the fight-flight-freeze response) in response to a trigger, similarly to how someone with post-traumatic stress disorder (PTSD) might respond to a trauma trigger. Curiously, misophonia trigger sounds are often most distressing when coming from a specific person, suggesting the disorder may be responsive to interpersonal relationship factors. Treatment of misophonia is currently limited to the use of hearing modifications (e.g., earplugs or headphones) and psychotherapy, but many patients continue to suffer despite these best efforts. Phase 3 clinical trials suggest that MDMA is efficacious at treating the symptoms of autonomic arousal, negative emotions, and interpersonal suffering found in PTSD. As such, we propose that MDMA may represent an ideal treatment for some suffering from severe misophonia. In this perspective article, we review the symptoms of misophonia, and outline how MDMA may be uniquely suited for treating it, perhaps using a protocol analogous to the MAPS Phase 3 studies for PTSD.

## Introduction

Misophonia is a disorder characterized by exaggerated, negative emotional, and physiological responses to otherwise benign sounds. The response to a trigger activates the autonomic nervous system in a “fight-flight-freeze” response (1), which is distinct from how most other people respond to sounds that annoy them (2).

The term misophonia was first used in 2001 (3), but as of this writing, has not yet been classified as a formal diagnosis in the DSM-V or ICD-11. Limiting issues so far include lack of a consistent definition and coherent diagnostic criteria for misophonia, although this is now being addressed through consensus work groups (2). Misophonia is a worldwide phenomenon that likely has been present in the human population for millenia, and is surprisingly common—around half of population samples have reported some degree of symptoms (4, 5), with up to 20% reporting symptoms that are clinically significant and may impact quality of life (6). Given how universal and prevalent this distressing phenomenon is, it is surprising that it has taken this long for the clinical and scientific community to identify it as a clinical entity, let alone begin formulating ways to treat it.

Treatments for misophonia are still scarce and mostly involve audiology interventions (e.g., white noise generating hearing aids) or variations of cognitive behavioral therapy (7). There are no controlled studies of medications yet, although case reports suggest that antidepressants (8–11), anxiolytics (12), stimulants (13), antipsychotics (14), and beta blockers (15) may help in some cases.

The biological mechanism of misophonia is slowly being elucidated (16, 17), and there are tantalizing clues that it shares overlap with the emotional and autonomic overreaction observed in other mental health disorders, such as post-traumatic stress disorder (PTSD), panic disorder, and specific phobias. Many so-called “misophones” react to a trigger sound analogously to how, for example, a soldier with PTSD after exposure to a bomb explosion might respond to hearing fireworks during a celebration, or how someone with a phobia of spiders may feel when encountering one. A flood of negative emotions and a surge in “fight-flight-freeze” sympathetic arousal is common to these examples.

An unusual feature of misophonia is that a trigger sound (such as chewing) is often much more distressing when generated by a specific person (7), usually someone emotionally close to the patient such as a parent or spouse. Other types of relationships, such as a new romantic interest, can have the opposite effect, and may reduce perceived distress from a trigger.

Trigger reactions to a sound are also more likely when the sound is felt by the patient to violate a rule or social norm. For example, the sound of loud bass drums at a concert may be perceived as acceptable or even enjoyable, but may become a disabling trigger if a next door neighbor is playing music at night, even if playing it quietly. An adult “rudely” smacking their lips may elicit uncontrollable rage, but this lip smacking may not be noticed when generated by an infant who is perceived as too young to “know any better” (7). This suggests that misophonia is not simply a reaction to the physical qualities of a sound, but that higher-order filters around relationship quality and social norms help modulate the perceived trigger distress, and may help determine whether the brain will activate a full fight-or-flight response.

MDMA is efficacious in treating disorders of autonomic arousal from negative stimuli. Recent Phase III trials (18), for instance, suggest that it might be a efficacious pharmacological treatment we currently have for PTSD. MDMA also increases empathetic, prosocial feelings toward others, and may be fairly unique in addressing negative emotions arising from interactions with others. These effects may address two core problems with misophonia: interpreting an otherwise benign triggering event as being very emotionally negative, and the inappropriate activation of a threat response (fight-or-flight) that typically comes with it.

In this perspective article, we examine the emotional and physiological features of misophonia and highlight the significant symptomatic overlap with other problems and disorders that MDMA appears to be efficacious at treating. We hope this can serve as a framework for future clinical trials for this common, sometimes disabling, and vastly understudied disorder.

## Method of literature review for this perspective article

We searched the literature (PubMed, Google Scholar) for any publication with the term “misophonia”. One hundred twenty results were returned, and were independently reviewed by both authors. The term “misophonia” yielded multiple reports that explored the characteristic emotional and physiological symptoms and proposed biological mechanisms underpinning misophonia. Of note, misophonia was not found in association with the terms “MDMA,” or any other dissociative or psychedelic treatments such as “ketamine”, “psychedelics,” or “psilocybin”.

To review the effects of MDMA on emotional and physiological symptoms relevant to misophonia, we focused on reports containing the term “misophonia” and any of the following: “anxiety,” “post-traumatic stress disorder/PTSD,” and “phobia.” To review effects on interpersonal relationships, we examined the terms: “empathy,” “relationship,” “mirror” (for mirror neurons), and “interpersonal.” We also examined effects of MDMA on the perception of negative stimuli, especially sound issues commonly associated with misophonia, and by searching the terms “auditory,” “phonophobia,” “tinnitus,” and “hyperacusis.”

In addition to search term reviews, references cited in recent reviews and meta-analyses were also examined, as were the “cited by” references in PubMed when examining key papers. We also searched https://clinicaltrials.gov/ for any future trails of misophonia and found four results, but none related to MDMA, psychedelics, or any other medication intervention.

The literature regarding the mechanism and treatment of PTSD and other anxiety disorders is vast, and far beyond the scope of this paper to fully capture. The study of the neurobiology of human relationships is similarly extensive, and beyond the scope of this work. As such, we examined the most recent reviews and meta-analyses on this subject (papers cited in Results section), to ensure an up-to-date accounting of the most recent mechanistic hypotheses, which were then compared to the literature regarding the mechanism of misophonia. Search limiters included all years, any language appearing in PubMed, and works published in peer-reviewed journals.

### Pathophysiology of misophonia

#### Auditory component of misophonia

Misophonia triggers are typically common sounds that do not cause excessive annoyance or distress to most people. They are often repetitive in nature, and most commonly generated by the mouth or nose of others. Chewing noises are prototypic, and are perhaps the most common trigger. That said, a vast number of different trigger sounds have been described, including non-human sounds such as dogs barking, water dripping, or the sound of percussion instruments. Of note, other sensory modalities can also serve as triggers and visual triggers (termed misokinesia) are also quite common. Sound triggers are the most extensively characterized, and are thus the focus for this perspective article.

Trigger sounds can lead to extreme distress, and are often compared to “the sound of nails on a chalkboard,” or for some, the feeling of being terrorized or physically assaulted. The distress from triggers can lead to impaired school or job performance, avoidance of certain normal life activities, social isolation, reduced life quality, and in extreme cases, self-harm (19).

Since misophonia involves a reaction to sound, it is often thought of as an auditory disorder, and frequently mentioned with other hearing problems such as tinnitus or hyperacusis (20). However, research now suggests that the peripheral auditory system, such as the tympanic membrane and auditory auditory nerve, is not the source of dysfunction in misophonia and that misophonia may not be related to other peripheral auditory problems. As seen in recent research by (21), misophonia appears to be a higher-order, central nervous system problem, rather than due to pathology of the peripheral auditory pathway.

#### Emotional response

Trigger sounds characteristically cause an unpleasant, aversive emotional response. Anger and rage has been the most commonly reported emotion, experienced by up to 90% of respondents in surveys (7, 22). Anxiety, panic, disgust, resentment, and self-loathing are other common emotions that can occur during a trigger.

After an acute trigger response is over, many misophones feel a sense of depression and guilt around how they responded, especially if they had hostile thoughts or actions toward someone. The intensity of these adverse emotions can sometimes lead to extreme feelings of hopelessness and even suicidal ideation. Some also experience repetitive, intrusive thoughts about the trigger and may continue to hear the sound repeat in their head, leading to continued physiological response symptoms. Anticipatory anxiety around future triggers is also common, and can lead to avoidance of situations that might cause a trigger.

In many cases, an adverse reaction to a trigger sound will only occur when produced by certain people (1). Because of this unusual phenomenon, misophonia patients will often develop intense negative thoughts and feelings about this other person. Resentment and hostility toward the trigger person is common, with an internal narrative that the other person must not care about them and is being intentionally rude, or is perhaps unacceptably oblivious to polite social manners that “should have” convinced them not to make the trigger sound. Many misophones recognize that these thoughts are irrational, and can vocalize this in an interview, yet will still find themselves having them during a trigger reaction, and even afterward (23). This dichotomy of caring about another person while still feeling a sense of hostility or avoidance toward them can be very distressing, and often convinces the misophone that something is terribly wrong with them. Many will “joke” about believing that they may have sociopathic traits, or worry that they may become “psychotic” or violent later in life.

In many cases, the hostile or avoidant reaction from the misophone leads to interpersonal relationship troubles. It is characteristically hard for many misophones to explain their symptoms to a partner or family member. The description of symptoms often sounds, to the partner, as an attack on them for something that should be relatively benign, such as the way they eat food. This can lead to anger, confusion, and resentment in both partners, and compensatory activities that further harm the relationship, such as if the patient will no longer eat meals with their partner.

#### Physical/autonomic response

Virtually all misophonic reactions to trigger sounds involve an immediate, involuntary autonomic response (1). The specific symptoms vary somewhat from person to person, but generally suggest a surge of sympathetic hormones leading to the so-called “fight-flight-freeze” response normally experienced in response to a dangerous threat. The symptoms also overlap considerably with someone exposed to a phobic threat, or during a panic attack.

Autonomic arousal happens within milliseconds of hearing a trigger sound, and feels like an overwhelming surge of emotion and bodily sensations that cannot be consciously controlled. Common symptoms include: elevated heart rate, increased respiratory rate, increased blood pressure, increased body temperature, sweating, clenched or tense muscles, and a pervasive sense of needing to fight the trigger or flee from it. Many misophones report being unable to think rationally when being triggered, and some will scream at or even hit the trigger source. They often feel as if their body is overtaken by the intense neurophysiological changes, so that their entire focus shifts to attending to the threat (trigger), to the exclusion of almost everything else around them.

This intense autonomic reaction to triggers is very different from the reaction the average person has to generally annoying sounds (2). Annoying sounds can produce measurable physiological effects in humans, but do not typically elicit a fight-flight-freeze trigger reaction normally seen when someone is intensely afraid or in grave danger.

#### Neurobiology of misophonia

Misophonia does not appear to arise from abnormalities in the peripheral auditory system as might be expected from hearing loss or tinnitus. Central auditory processing, on the other hand, was found to be abnormal for misophonia patients as measured by N1 auditory evoked potentials to oddball stimuli (24). Functional neuroimaging has also shown overactivity in the auditory cortex in misophones compared to controls (25, 26).

But these differences in central auditory processing do not fully explain the symptom constellation observed in misophonia, such as why highly specific, complex sounds are triggering, as opposed to types of sound with a certain audiological certain quality such as those that are too loud or high pitched, which we might expect from a peripheral auditory hypersensitivity. Auditory processing issues also do not explain why trigger reactions seem to arise preferentially from certain people or only in certain social or environmental contexts. And as noted, the intensity of the reaction to triggers is unlike the reaction to regular annoying sounds, and more resembles a reaction to life-threatening danger. These symptoms suggest unique abnormalities in brain regions connecting sensory input to higher-order emotional processing and threat recognition centers.

Changes in those with misophonia have indeed been observed in regions that mediate connection between perception of stimuli and emotions, in particular, increased activation of the right anterior insular cortex (AIC), a core part of the salience network (25, 27). Abnormal connections between the AIC and the posteromedial cortex (PMC), ventromedial prefrontal cortex (vmPFC), amygdala, and hippocampus were also observed. The AIC is critical for managing focus and attention, and ensuring someone can devote the needed mental resources to processing a complex task. In line with these observations, many misophones describe trigger sounds as being mentally intrusive (1). Studies similarly show that trigger sounds disrupt attention and focus in misophonia patients, when compared to disruption from simply hearing an annoying sound (28). This suggests that misophonia triggers are not simply “annoying sounds” in the conventional sense, but may be causing a deeper sense of cognitive disruption. Being interrupted while trying to focus is commonly experienced as irritation and anger, which are also emotions commonly experienced in response to a misophonia trigger.

Positron Emission Tomography scans have revealed that MDMA decreases blood flow to the motor and somatosensory cortex (29), which are regions important to the motor sensory integration component of misophonia. Blood decreases were also observed in regions central to negative emotional processing, including temporal lobe, left amygdala, cingulate cortex, right anterior insula and thalamus (29, 30). This may suggest a reduction in activity of these emotional centers involved in overactive threat responses, such as those suffering from PTSD and anxiety (31).

Misophonia seems to involve overlapping brain regions also affected by MDMA, including the left amygdala, anterior insular cortex, and anterior cingulate cortex (25–27, 32). Misophones also seem to exhibit overactivation of their left amygdala and temporal lobe in direct response to auditory triggers (32). Specifically, results showed hyperactivity in the bilateral superior temporal cortex during triggering episodes in misophones. Increased activity in the left amygdala could also explain the hypervigilance that is seen in misophones when exposed to particular triggers (32).

A breakthrough in mechanistic thought about misophonia is that it may be due, at least in part, to excessive activity of the brain’s motor system and mirror neurons. Mirror neurons recognize complex behaviors and activity sequences in others (26). This system is thought to be crucial for understanding the actions, intentions, and emotions of others (33–35), and may play a key role in socializing and having empathy for others (36).

At a more basic level, when mirror neurons recognize an activity sequence in someone else (such as chewing food), the motor cortex activates as if the observer was actually doing that activity. It has been proposed that in misophonia, these mirror neurons may be “hyper-responding” to activities associated with a trigger, and that this hyper-response causes distress (26).

The mechanism of misophonia appears to be quite complex, and involves aspects of cortical auditory processing, changes in attention and focus, aberrant negative emotional interpretation of a stimulus, and a corresponding physiological threat response. It will likely take many years before it is completely understood, but attempts to develop symptomatic treatments need not wait this long. Misophonic reactions to triggers share considerable symptom overlap with other disorders that are better understood, and that have already powerfully responded to treatments such as MDMA.

#### Similarities of misophonia to post-traumatic stress disorder

The misophonic response to triggers shares considerable symptom overlap with sound trigger responses observed in PTSD. As with misophonia, those suffering from PTSD can experience a very strong emotional and physiological response to specific stimuli (such as sounds) that do not bother most people. For example, someone who was abused may be triggered by the sound of a voice that resembles the attacker.

Post-traumatic stress disorder responses to triggers are mediated in part by a “fight-flight-freeze” autonomic surge, which can cause increased heart rate and blood pressure, sweating, hyper-vigilance, feelings of anger or aggression, and inability to think rationally, outside of an intense focus on fighting or fleeing the situation. Autonomic hyperarousal is a risk factor for developing PTSD symptoms (37), and those suffering from PTSD tend to have increased baseline sympathetic nerve activity (38). These autonomic symptoms bear a striking resemblance to those often observed in misophonia, and notably, PTSD was found to be a common comorbid condition with misophonia (39). This may speak to common underlying neurobiological traits and vulnerabilities for both disorders.

It is important to note that although there are similarities between PTSD and misophonia, there are also many fundamental differences in these two disorders as well. PTSD is specifically defined as having an automatic strong reactivity to a spontaneous, involuntary, and intrusive distressing memory of a terrifying traumatic event (39). While in misophonia, the strong negative emotional reactivity is triggered by seemingly harmless stimuli only. PTSD and misophonia are two very distinct disorders that also happen to share similar symptom profiles and neurobiological mechanisms.

PTSD also features other symptoms not typical of misophonia, such as intrusive recall of the trauma, emotional numbing, an exaggerated startle response, and frequent flashbacks. These differing symptoms clearly highlight how these are different disorders, just as PTSD is also not the same thing as, say, a specific phobia. That said, the strong overlap of inappropriate autonomic activation in both offers clues into what medication treatments might be useful. Medications that reduce autonomic hyperactivity can reduce many of the distressing symptoms of PTSD (40). And intriguingly, there are emerging observations that medications that reduce autonomic response may also be helpful in some cases of misophonia (15).

From a neurobiological perspective, a hyperactive amygdala is observed in PTSD (41), and similarly, an enlarged and overactive amygdala is also observed in misophonia (17). Abnormal connectivity between the insula is also observed in both misophonia and PTSD (42). While there are some general functional central nervous system (CNS) similarities with PTSD and misophonia, the complexities of measuring brain activity in response to stressors is complex and relies on numerous environmental and study population variables particular to each study. As such, a direct comparison under similar experimental circumstances will be needed to more definitively compare CNS activity between PTSD and misophonia.

#### Similarities of misophonia to panic and phobias

While anger is the most common emotion experienced during a trigger, anxiety, and panic are also very common. The physiological symptoms of intense anxiety, phobia, and panic attacks are very similar to those experienced in response to a misophonia trigger; and panic disorder and anxiety also frequently co-occur in those suffering from misophonia (43). This may suggest some common mechanisms and vulnerabilities.

It is worth noting that misophones generally do not feel afraid of their triggers (1). As such, the misophonic response is not simply a phobia of sounds, which is a separate disorder termed phonophobia. Phonophobia is more specifically a fear or intolerance to sounds, based on specific physical characteristics of sound such as volume and pitch. It usually arises secondary to an illness or defect in the peripheral auditory pathway that causes an unpleasant sensitization to the sound, resulting in the patient becoming afraid or avoidant of sounds with those qualities (44). This is different from misophonia, which is a highly specific aversion to often quiet, non-threatening sounds that do not appear to be aversive based on their physical qualities.

#### Misophonia and other psychiatric disorders

Other psychiatric disorders, such as depression, OCD, and anorexia can co-occur in misophonia, and may moderate the severity of the symptoms (39). The wide variety of disorders that may be associated with, and may affect, the experience of misophonia highlights how much we have yet to learn about it.

## Proposed MDMA effects on relevant misophonia symptoms

There are no formal studies on the use of MDMA to treat misophonia, and so this proposal stems from theoretical observations of how MDMA works.

### MDMA effects on perception of negative external stimuli (e.g., sounds)

The process of being triggered from a sound likely involves, to some extent, abnormal auditory processing. Importantly, MDMA can affect the perception of sound. Animal models show that auditory sensory gating is directly affected during MDMA use (45), and that this action depends on the serotonin receptor 5-HT2A (46). Since auditory gating may also be affected in misophonia (24), it would be of interest to see if MDMA administration could counteract these auditory gating abnormalities, but this remains to be examined.

A study in healthy recreational MDMA users showed that 75 mg reduced autonomic arousal to negative/unpleasant sounds, independent of the overall level of arousal (47). The authors specifically noted that clients “felt less discomfort or more at ease” when exposed to unpleasant sounds. While this study did not specifically address patients misophonia, this clear reduction both in the subjective feeling *and* autonomic response to sound offers particular hope that MDMA may also be useful for aversive autonomic reaction toward misophonic triggers.

In a broader sense, MDMA is well known to reduce the perception of negative visual and other emotional stimuli, such as the recognition of sad, angry, or fearful facial expressions in others (48, 49). It is particularly effecacious at inhibiting a fearful or avoidant response to such stimuli, while allowing the user to maintain rational cognitive control, such as during a psychotherapy session. The mechanism for this is not clear, but some have noted that since MDMA decreases cortisol levels and amygdala activation, which may allow the patient to better access difficult, emotional material in the session. At the same time, MDMA may increase oxytocin levels (31), allowing for stronger bonding with the therapist and/or partner during the therapy session.

In rodent models, MDMA may also modulate how fearful or unpleasant memories are recalled, again allowing for a calmer, safer approach to processing them during a therapy session (50). It may also improve the extinction of any aversive emotions attached to unpleasant memories when doing exposure-based therapy (51, 52), although the degree to which this happens is disputed (53). This impact on prior learned emotional responses may offer a way to also re-imagine the emotions attached to triggers, and to the people who produce those triggers.

### MDMA effects on disorders of autonomic hyperarousal (PTSD)

Misophonia triggers induce a strong autonomic response that mediates much of the physical symptoms, and can reinforce the emotional perception of the trigger as being aversive, analogous to the strong autonomic symptoms experienced during a phobia reaction, panic attack, or a PTSD trigger. A pharmacological agent that could reduce this strong negative, autonomic response may thus potentially be an effective treatment.

Numerous reviews and meta-analyses consistently support MDMA as efficacious for treating PTSD symptoms, even long after the acute effects of the drug (40, 54–60). These clinical benefits appear to exceed any offered by current first-line pharmacological treatments (61). A recently completed Phase III clinical trial (18) has similarly shown good efficacy for PTSD, and therefore, MDMA may soon be offered as a mainstream treatment for it. MDMA is also showing promise for treating other anxiety-spectrum disorders, particularly social anxiety (62). The effects on other disorders such as phobias and panic are unclear, and need more study.

### MDMA effects on rigidity and internal ruminations

MDMA and other entactogens have a unique ability to promote cognitive flexibility, and help patients get unstuck from negative thinking patterns that trap them in cycles of depression, anxiety, and PTSD. Patients will sometimes report feeling able to see a problem with a brand new perspective and see more helpful ways to reframe it, or perhaps be able to forgive and close a chapter on a painful topic. Indeed, negative memories are rated as less negative by subjects taking MDMA, while positive memories are remembered more vividly (63).

While external stimuli (triggers) are the main immediate source of distress in misophonia, internal negative thoughts and feelings may also reinforce or aggravate the disorder, and perhaps sensitize the patient to have a stronger negative reaction toward future triggers.

The sounds that become triggers also often associate with activities seen as violating a social rule or norm. For instance, a patient may develop a trigger reaction to family members eating with their mouth open, when it has been implied in that family that eating with your mouth open is rude. Hearing thumping music being played late at night violates the generally accepted social norm of not playing loud music after hours. While many people can get annoyed at late night loud music, for the misophone, this dislike may grow over time into an intense, triggering rage even when the music is not overly loud or rude.

Misophonia sufferers have high levels of neuroticism (64), and anecdotally, often report that a more rigid insistence of rules and social norms may make them less tolerant of sounds and behaviors that may seem to violate these. Addressing neurotic personality traits is difficult and usually requires years of therapy, often with incomplete results. MDMA can increase feelings that run counter to a self-critical, neurotic state, namely: self-compassion, empathy, forgiveness, openness, and reduced feelings of conflict (65–67). This may open a window of cognitive flexibility to challenge how “rude” or “inappropriate” a trigger sound really is. If a trigger sound could be reframed as something reasonable and acceptable, the distress around it may be reduced.

### MDMA effects on relationship quality

A very unusual characteristic of misophonia trigger sounds is that they are often localized to specific people. Chewing sounds from friends may elicit no reaction, while the same chewing sounds from a parent may be intolerable. Certain kinds of emotive, new highly desirable relationships (e.g., romantic), are anecdotally reported to be protective against trigger effects. This relationship specificity suggests that the interpersonal, emotional context of a trigger may also play a role in determining its severity. If this is the case, then changing the emotions a misophone feels toward a trigger person may reduce the trigger distress.

MDMA is one of the few pharmacological agents that can profoundly change the feelings people have toward each other, even long after the acute effects have worn off. Couples who use MDMA often report sustained, reduced conflict, increased empathy for each other, happiness, feelings of closeness, communicativeness, generosity, attachment safety, bonding, and intimacy (65, 68, 69). MDMA significantly increased the perceived pleasantness of human to human slow touch, but did increase perceived pleasantness of fast touch (70, 71).

The prosocial effects of MDMA were found to occur among couples in which one was suffering from prior trauma (69). MDMA thus appears to be a promising pharmacological treatment for couples undergoing therapy (Cognitive Behavioral Conjoint Therapy) when one partner has PTSD, with high overall satisfaction rates for both partners (72).

### The biological mechanism of MDMA effects on misophonia

It has been previously noted that MDMA increases serotonin, norepinephrine, and dopamine activity by stimulating neurotransmitter release and inhibiting neurotransmitter reuptake in the synaptic cleft (73). This is thought to be part of the reason for the positive, euphoric effects. MDMA also directly binds to and stimulates serotonin 2A receptors (5HT2AR) (74), and many of MDMA’s effects on anxiety, fear, and processing of emotions appear dependent on normal functioning of this receptor. MDMA also improves the results of exposure-based therapy for PTSD, perhaps due to enhancement of fear memory extinction, although some studies dispute this (50). In animal models, blocking the 5-HT transporter (5-HTT) eliminated fear memory extinction effects (52), again highlighting the importance of the 5-HTT and 5-HT2A receptors.

Of critical importance to misophonia, 5-HT2A receptors appear to also be critical for MDMA’s ability to reduce arousal caused by negative sounds (47). However, MDMA also affects numerous other neurotransmitters, hormones, and other gene regulators critical to homeostasis and emotional regulation, such as glutamate, oxytocin, cortisol, vasopressin, adrenocorticotropic hormone (ACTH), prolactin, and brain-derived neurotrophic factor (BDNF) (51, 75–77).

Looking even more broadly, other biological systems related to inflammation and immune regulation may also help mediate the effects of MDMA (78). Whole-genome microarrays in animal models exposed to MDMA show *hundreds* of gene expression changes from a variety of different cellular processes (79). These changes vary depending on location in the brain, concurrent environmental cues, and also no doubt will vary depending on the animal model used (none have yet been done in humans). As such, it is clear that a comprehensive molecular understanding of how MDMA effects change is still some distance away.

Meantime, regardless of how MDMA actually produces its neuropsychiatric effects, we should not lose sight of how beneficial it can be in treating disorders that are otherwise notoriously difficult to help. Thus, the development of treatment protocols should not wait on certain determination of the underlying mechanism.

## Discussion and proposed treatment protocol

MDMA offers a unique constellation of benefits that appears almost custom-fit for the symptoms commonly encountered in misophonia. It directly reduces autonomic reaction to aversive sounds, and shows promise for treating disorders characterized by abnormal autonomic arousal (e.g., PTSD). MDMA reduces the fear and avoidance while promoting openness, acceptance, and empathy, which may open a new window during psychotherapy sessions to retrain the negative response to misophonia triggers.

MDMA also appears to uniquely address neurotic ruminations while also reducing negative feelings that underpin interpersonal conflict. We anticipate some cases in which a patient in assistive therapy will realize that they were subconsciously holding on to social norms, neuroses, or resentments that are aggravating the emotional and autonomic response to triggers. The neuroplastic state induced by MDMA may allow deeper ruminative underpinnings triggers to be consciously identified, and perhaps laid to rest.

The ability of MDMA to facilitate healing in couples in which one member suffers from PTSD may be particularly germane to misophonia. As with PTSD, the patient with misophonia often suffers from abnormal emotional and physiological responses to the partner, and the partner also suffers as a result of these abnormal reactions. The beneficial effect of MDMA on both people may thus offer a unique approach to severe cases of misophonia in which both partners are suffering.

### Risks of using MDMA for misophonia

MDMA is generally safe and well tolerated when used in a therapeutic setting, and most side effects occurred during acute treatment, and were transient. In a large study in healthy volunteers (80), side effects included a temporary increase in blood pressure, tachycardia, and increased body temperature in about 1/3 of participants, with no serious adverse effects. A meta-analysis of MDMA for treating PTSD (54) also found that therapy was generally safe, but teeth grinding, anxiety, headache, nausea, and feeling “jittery” were common during acute intoxication. One of the most serious theoretical concerns (serotonin syndrome), is based on MDMA’s ability to increase serotonergic activity, but so far no cases have been reported in clinical studies (81). Not all experience MDMA as pleasant and instead may feel anxiety and panic during a session (82), and thus it is necessary to counsel clients ahead of time that it is possible they will not enjoy or benefit from the experience. It is also possible that MDMA will not help some cases of misophonia, which could be disappointing, particularly since there are few other pharmacological alternatives at this time.

It is critical to understand that recreational use of MDMA is NOT recommended at all. This is a powerful treatment that must be administered in a safe setting, by a trained medical and psychological professional. The concentration and purity of MDMA used outside of research settings cannot be validated since this still remains a schedule 1 substance. Consuming MDMA recreationally may involve risks such as ingesting impure substances, ingesting excess amounts, and experiencing unpleasant effects in unsafe environments. Before recommending MDMA to people struggling from misophonia, randomized controlled trials with good generalizability are needed for evaluating the effectiveness of this treatment.

### Proposed model for treatment of misophonia with MDMA-assisted therapy

Based on the analogy of misophonia trigger symptoms with PTSD, it seems likely that MDMA is a efficacious pharmacological treatment for PTSD, and may also be of benefit in misophonia, both for individuals suffering, and for couples who are both affected as they often are in PTSD (69). Given the similarity of symptoms, we see no reason to deviate from the protocol used in Multidisciplinary Association for Psychedelic Studies (MAPS) Phase 3 studies.

MAPS is one of the leading organizations investigating the use of MDMA-assisted psychotherapy in treating disorders such as PTSD. Their current Phase 3 PTSD protocol (83) has used 75–125 mg of oral MDMA, given during standardized psychotherapy sessions for PTSD in 2–3 total sessions each spaced about a month apart. Three preparatory psychotherapy sessions without MDMA were administered prior to the dosing sessions to build rapport and set goals, and 3–4 sessions would follow, for a total of 8–10 sessions.

During MDMA treatment, feelings and emotions around triggers could be explored in a manner similar to that done for other unpleasant subjects such as trauma. The therapists could assist the client in examining when and how they react to a trigger, and if they wished to consider examining whether they should be more open and accepting toward those making the trigger sounds. It may also be possible to do real-time exposure work to triggers, since MDMA can reduce the otherwise overpowering aversive response that could interfere with the therapy.

As with the PTSD studies, it will be critically important to have the 3–4 follow up sessions in order to integrate the experience, build skills around what was learned, and help any clients that may not have experienced sufficient benefit from the experience. In many cases, it will likely be appropriate to continue regular psychotherapy and medication management well beyond the 8–10 session series, in order to reinforce new habits and ways of responding to things.

In terms of patient selection and inclusion criteria, we would advise that adult patients be selected who have misophonia according to the 2022 consensus definition (2), and that score in clinically concerning range on a generally accepted misophonia rating scale, so that symptom reduction can be more systematically tracked over the course of treatment. Historically, the most widely used rating scale has been the Misophonia Assessment Questionnaire (Johnson, available online, however this is slowly falling out of favor. Newer rating scales such as the S-Five: Selective Sound Sensitivity Syndrome Study (84), Misophonia Response Scale (85), the A-MISO-S (4), or the now revised version AMISOS-R (7), the Duke Misophonia Questionnaire (86), and MisoQuest (87) have made progress toward having a fully validated, internally consistent misophonia rating instrument, and may also be a good choice for tracking progress.

Exclusions for participation would include all comorbid disorders. To establish promising and accurate results on the effectiveness of MDMA on misophonia, it is important to control for all of the possible confounding factors, including pre-existing comorbid disorders. In future clinical practice, we anticipate that misophonia and PTSD will be frequently treated together and see no reason to oppose this, but for purposes of an initial study, would advise these be kept separate. Other exclusions would be any medical contraindication for receiving MDMA, concurrent Bipolar 1 disorder, borderline personality disorder, eating disorder with active purging, or active substance abuse.

Given the highly relational nature of some misophonia sufferers, it would also be advisable to develop protocols for a separate couples therapy study, similar to the successful couples work in which one member suffers from PTSD (69). Empathy, understanding, and forgiveness related to misophonia triggers between the couple could also be an area of focus.

In terms of risk to participants compared to the current treatment protocol (i.e., for PTSD), we note again that misophonia sufferers have high levels of neuroticism (64), and that neuroticism corresponds to a higher risk (82) of having an unpleasant experience with MDMA. This could mean that a higher proportion of misophones will experience a negative experience on MDMA compared to the general population, and this may be a limitation to these studies.

## Conclusion

MDMA offers a unique profile of emotional and physiological benefits that may be particularly helpful for treating misophonia, including: reduced arousal from hearing aversive sounds, reduced negative emotions toward an undesirable event, a greater sense of openness, acceptance, and forgiveness, and an increase in emphatic, prosocial feelings between those who may otherwise be in conflict. MDMA has already proven itself safe and efficacious in other similar disorders characterized by aberrant emotional and autonomic responses, and we propose it is ready to also be tested on misophonia symptoms, with a protocol analogous to the MAPS Phase 3 trials for PTSD. A successful outcome would represent a major breakthrough in treating this common and complex disorder, which currently has limited treatment options.

## Data availability statement

The original contributions presented in this study are included in the article, further inquiries can be directed to the corresponding author.

## Author contributions

JW: background research, conceptual development, and writing of the manuscript. SK: background research and writing of the manuscript. Both authors contributed to the article and approved the submitted version.
